# Supplementary material for: Using fatherhood to engage men in HIV services via maternal, neonatal and child health entry points in South Africa
Source: PLoS One. 2024 Jun 27;19(6):e0296955. doi: 10.1371/journal.pone.0296955 (PMC11210759; doi:10.1371/journal.pone.0296955)
Supplement: S1 File — (PDF) [file pone.0296955.s001.pdf]

**Project Name:**	Fatherhood & MNCH
**Site:**	
**Record Number:**	FGD_02_040621
**Transcribed by:**	
**Used language(s):**	
**Interviewed date:**	04/06/21
**Transcription date:**	
**Duration of the interview:**	
**Checked by:**	
**Checked date:**	
**Edited By:**	

INTERVIEWER: Thank you so much gentlemen for taking your time with us, we appreciate your availability. As we mentioned earlier on there is no right or wrong answer, we would like to understand your opinions in terms of how men experience fatherhood. The study aims to improve the involvement of fathers in their partner's pregnancy and the early years of childhood. When someone is speaking, please give them a chance to finish his opinion. Please always start with your identifier when you answer the question. If you did not understand the question, you can ask us to repeat it. So, I will ask the first question whoever thinks has an answer can raise their hand and I will point you to speak. We are also recording as we mentioned earlier, please give each other a chance to speak so that we can be able to hear the recording properly later.

INTERVIEWER: What does it mean to you to be a father? [Probe different fatherhood: absent, social, involved, etc]

RESPONDENT 12: Being a father means that you need to know who you are, and what you stand for in your family.

RESPONDENT 7: I think being a father means that taking full responsibility of your family,

RESPONDENT2: being a father means that being a good example to your children.

RESPONDENT9: Being a father is an exciting thing to us as men that we will have a child, but at the same time you need to understand that you will be their shield in everything that might will happen in their life, you need to understand that their look up to you. And you must lead them.

RESPONDENT08: in my understanding, being a father does not necessarily mean you need to have kids of your own, you can be a father to your nephews and nieces, and as long as people look at you as the father figure it is enough.

RESPONDENT 03: being a father means respecting the mother of your children, that woman is too special to give you your firstborn. Then your role comes after you have impregnated her as the father,

now you respect her as she has made you a father on the earth, you thank your woman that she is going to make you a father. Although sometimes women make you raise a child that is not yours but also thank her because she has given you a chance to be a father.

RESPONDENT 10: Being a father means that you understand your responsibility and that you have brought someone on earth now you need to act like a man and be a good example to your children.

INTERVIEWER: How do you think other people understand the idea of fatherhood or being a father?

RESPONDENT 06: I think it is their actions, that determines what kind of father you are, or how you will look after your children.

RESPONDENT03: guys, we know, as gents we know, we have friends and brothers some guys think that being a father is about money, they only provide financial support to their kids. For instance, they know that I have a child with Zanele, and we have agreed that I will give her R3000. 00 monthly and that's all and you give Zanele that money and it ends there. There is a guy who doesn't have anything who hustles and will meet up with his child in the street and he would say come here my boy have this R3 buy chips, being a father means you must be present in the life of the child, let's forget about the money, yes, we cannot take away the power of a relationship with the mother and the child, as men our challenge is being present in the lives of our children. If you are a father, you should not be useless to the point that your child will say this is not my father, remember he is a person also as much as he is a child, he is also a person.

RESPONDENT10: okay, other people look at being a father as a part-time job, for instance, some people know that there have 6 children, but they are only taking care of 2 the other 4 they do not know anything about them, as P03 has said as men we only think being a father means you need to be there financially forgetting about the relationship that one needs to have with their children. As the result, there are many of the gents who are bitter and have no direction because they did not have a father figure to give direction while growing up which ends up being a chain in our society.

RESPONDENT 7: What I have noticed is that most gents, have kids all over the world and you find that person will make a joke about having 7 kids when we are chilling as gents and that person is not even taking care of his children, you find them telling us in the Taverns that they have kids, but they are not even doing the right thing to their kids. And you find out that there are guys out there who want to have kids, but the unfortunate part is that they are not blessed with the kids.

RESPONDENT 03: I think other people think that being a father is being determined by what you wear, having a beard, this and that which might mean nothing, if you are not responsible to your family, you are not a good example to your children but for the fact that he wears the trouser then he thinks he is a father, yes a father needs to have his way of doing things and be sure that his family is taken care of especially the children and instilling good values to your family. So, some fathers think that being a father is about being old and having kids without attaching the responsibility to it.

INTERVIEWER: How do you think fatherhood has changed over time in South Africa? What is contributing to these changes? Are you aware of any new fatherhood styles that are being practiced in South Africa? If yes, please describe them to the group.

RESPONDENT 02: I think that the rights of the fathers are not taken seriously as compared to mothers. Some fathers wish they can have full access to their children but because of the right of women being superior to those of a man, it becomes difficult. Because as a father you have 10% right over own biological children as compared to mothers who have 90%.

RESPONDENT 09: Us the modern fathers, vs the olden fathers, the modern fathers have changed a lot from the old fathers, you know I think the modern fathers try so much to be free with their kids which sometimes has a negative impact on the kids. For instance, I am the person who smokes weed and our children copy a lot from us, I have an 18-year-old son who is already smoking weed, also with the old generation fathers, we used to copy a lot from our fathers, for instance, I used to copy what my dad will do when I was with him, let's say he sat down, I will also want to seat like him when he drinks something I will want to drink also. Some fathers have been raised by women only because their fathers were not there and they were working, so the fathering style has evolved because of work and because of the society we live in but what I know is that we as modern fathers we are lacking a lot as compared to the old generation.

INTERVIEWER: what is it that you think as modern fathers you are lacking?

RESPONDENT 09: I am not sure what can be done to honestly help us, but I think coming together as fathers and realizing that we are not doing right by our children.

RESPONDENT05: I think I am the youngest in this group, my son is 3 years, but when p09 says that we are lacking as modern fathers I think that is not true, myself as the father I always tell myself that I do not want my kids to grow and experience the same things I have gone through in life when I was young. I grew up without my parents, my mother passed away I think I was 16 years old; my dad when I was 6 years. Us as fathers, where we lack most is taking responsibility, we impregnate women and leave them. All children are known by their mothers, as fathers, we lack one thing which is the responsibility, money does not raise children, children are raised with love. Myself, I sometimes sit on the couch, and my boy just comes and lies to me, it is my son, he just wants to sleep on me, and we as modern fathers we do not have the time to give our children that love. The only thing that is affecting us so much as the modern generation is that we are all at the same level, we want 50/50. There are families where you will find out that the wife is earning more than the husband, but you will not see her disrespecting the head of the family. For instance, you will find out that you will go ask for money from your father and your father will tell you to go ask your mom because of the communication and the respect they have, the mother gives the father the respect he deserves therefore that house will be in peace.

INTERVIEWER: **If you chose to have your child(ren) – why did you want a child/child?**

RESPONDENT 04: I wanted to have kids because I wanted my bloodline not to end, I wanted to increase my surname.

RESPONDENT 05: Honestly speaking, I did not want to have a child, number one I am not financially stable, which means I do not have a house of my own, and I have mentioned before that I do not want my child to grow up like me, I want my kids to grow up having their own home. So, most women have power over when they want a child and with whom, you will date a person for 2-3 years straight and she decided to stop the injection and opt for maybe pills, now the pills they forget them and once forgotten the chances of being pregnant are very high. Sometimes we choose if we want to have a kid, but if our finances are not good then we do not have an option for the child.

RESPONDENT 13: in my view, I do not think we plan to have kids but what happens is that it happens unexpectedly and once it happens it's a blessing from, God.

INTERVIEWER: If you did not plan/actively choose to have a child/child, how did you feel when you found out your partner was expecting a child?

RESPONDENT: as men we do not plan that, I remember we agreed with my wife that yes, we can have another one, but the child did not come up until the time we were not looking for one.

RESPONDENT 03: What happened to me is that I dated my girlfriend in 2003, my friend dated his girlfriend from 1999 when I first got my firstborn in 2006, my friend asked me how come because they have been trying how I did it, I told him I do not know because even myself I did not know my girlfriend came to me as said she is pregnant. So, this thing happens on its own, there is no planning for us as men, so my friend was worried that he might not be having kids because he has been trying since 1999.

INTERVIEWER: How did you feel when you found out your partner was expecting a child?

RESPONDENT 06: I did not plan all my kids.

INTERVIEWER: The time your girlfriend said I am expecting a child how did you feel?

RESPONDENT 06: It's scary because you know that you live for someone, you need to change all your ways.

RESPONDENT 05: you can all disagree but you know what I am talking about when a woman says I am pregnant, the whole 9 months you have doubts that it's your child until the child is born and you hold him with your own hands and now you see the resemblance, but the whole 9 months whether you are married or not but the whole time there are always doubts running up and down in your mind. You will be thinking that it might not be your child.

RESPONDENT 12: for myself, it was a challenge because I did not believe I can have a child, but as P05 said no man believes that the child is his until they see and hold the child.

RESPONDENT 10: with the feeling of going to be a father, getting a message from the mother, is the important phase for me because I become happy, now when the child is about to be born that is when you start stressing out and start looking at the ones that you have you look at your firstborn and wants something that will give you a surety that it's your child, now when the new one is about to be born you already have features that you want to compare with the newborn. You are not going to ask the mother directly whether this is your child or not, that makes a good father. You should be able to see it yourself.

Fathering experiences

INTERVIEWER: Can you please share with us any life experiences and relationships you have had that have impacted your own fathering (whether currently actively involved or not), for example, your own father/father figures, your partner, your partner's family, religious leader?

RESPONDENT 3: To be the father that I am today, it is because of the mother of my kids, gents, if the mother takes care of her children that makes you a good father, as I have mentioned that if I am left with my kids, they see a cartoon, because my wife, the way she looks after her kids makes me be a proud father. In all honest fact, we are not the same when it comes to gender roles, we as men are "dogs", women are snakes, but I have gained a good snake that can make a house a home, the way she looks after my kids I am so proud of her, she takes care of them in a manner that I do not think I can match what she does. If you get a woman who loves her children it makes your heart the father be at ease. If the woman loves her children, then there is no work that the father needs to do.

RESPONDENT 07: I know how I grew up, I grew up with my great grandmother, so I decided that I will not allow my kids to grow up the same way as, to be specific not staying with my biological parents I didn't like it, so I told myself that even if we are separated with their mother, but I will be present in their lives.

RESPONDENT 06: So far with my children, I am trying to be everything they need, a friend, a brother, and a father because I have seen that most kids lose direction because of not being taken care of by their fathers, so in my experience, I grew up with both parents, but my father was a very strict man.

RESPONDENT 13: I was never raised by a father, when I grew up I had no father figure, I was raised by women, so at an early stage I had a girlfriend and impregnated her, I was so stressed out and all, that is when I started seeing that I am no longer a boy, now I am a man, I need to be responsible and I started having my financial problems because I had to take care of the mother of the baby and the baby.

INTERVIEWER: In all that you have mentioned, is there anything that you remember that has shaped you to be the father that you are today?

RESPONDENT: I think there are different things I have learned from different people that have helped me to be the man that I am today, I have met different women that I loved and they loved me too, but because I was not ready for the commitment and I was still young according to me so now I am ready for the commitment then things go south, but all in all I have adopted things as I grew up around people I loved and cared about.

RESPONDENT 05: okay, so apparently, not so long ago I found out that my dad was not a responsible person, at home, for instance, if they play with other kids, I teach my boy not to be bullied at home or anywhere, I teach him to be a soldier and I am not going to let anyone bully my kid. I teach him to be able to stand up for himself when I am not there, when I see someone pushing him, I do not say apologies I tell him to push back, the way I grew up, and the things I have seen on the streets, and how life has tortured me, it has changed me to be the father I am today. So as the black family we have been raised to succumb in situations that make us shrink and not be able to speak out.

INTERVIEWER: What is your current role(s) in the raising of your children (biological or non-biological)? In what ways as a father are you physically, emotionally, financially present in the lives of your children? If you support both biological and non-biological children, do the support you give the same or different. If different, please describe the differences and why?

RESPONDENT3: In my street, there are many kids, and I always play with them, I am a person who loves kids, I call the young boys soldiers and they call me soldier, now on the little girls I call them Queens, now they become confused because when they say soldiers I say Queen now I am not sure what I create in their minds because they do not know the difference between the Queen and the Soldier. But I am trying to create an army that has queens and Soldiers. My explanation of this is that when we live together let's live in harmony, lets create that togetherness within our kids so that they will grow up loving and respecting each other, let's not fight and I'm teaching boys that when they see a woman, they must see them as queen.

RESPONDENT 07: I think the role I have played in my child is very important you know what happened, at school, they were asked about their role model and everyone in the class were calling for different people who are celebrities, such as Michael Jackson, Nelson Mandela but my son said I am his role model. When I heard that I was so happy that there is something beautiful that I am doing in front of him, that he looks at me as one of the people who has value in his life.

INTERVIEWER: Which of these elements (physical, emotional, financial presence) is important for you? Why in terms of raising your kids?
RESPONDENT 02: I think the important thing is teaching our kids how to do the right thing all the time, teach them to love education and stay away from trouble, I always tell mine that they need to love

education because these days it is rare that you can become something great if you do not study and stay away from drugs. Another important thing especially for the girls, they need to be kept close and be told that they are loved to avoid that hate that children sometimes grow up with when they think that their fathers do not love them. So, I think education is what we should teach our kids.

INTERVIEWER: do you stay with your kids?

RESPONDENT02: Yes, I do stay with them not all of them though.

INTERVIEWER: **Okay, so how do you support yours, emotionally, financially, and do you speak to them and tell them about how important is education?**

RESPONDENT 02: Yes, you see my sister, I am not working but I make sure that I make my kids happy. You can go ask them if they ever go to bed on an empty stomach, they don't get grants, but they will tell you that our dad makes sure that we have food. You know what I am telling you I have also experienced it when I grew up, I did not stay with my father he is absent in my life, but it was someone I knew but he did not care about us. So, what I told myself is that let me push in life because I do not want my kids to go through the same thing.

RESPONDENT05: the role that I play in my child every day, except on weekends, so what happened is that before my child came to permanently stay with me, he was staying with his mom. Every month there will be things that are short and finished before the month-end until the Covid-19 times, so I asked her to give me my son because when he is with me if I do not have money at home, they will be able to assist, and he can eat what we are eating. You know my son is like a brother to me, I am there financially, emotionally, and physically.

RESPONDENT12: The truth is that I do not do the same in all my kids, this is because the ones that I stay with are not my biological kids, my biological kids I do not stay with them. The reason for not doing the same for all of them is that with the ones I stay with it is easy to see what they need rather than those that I do not see every day. So, with the ones I do not stay with, I sometimes get the notification when I do not have money, and only sending them later.

RESPONDENT07: With me, they are my brother's children, now with my brother, it is not that he is not looking after them he does whenever, so his kids are my kids, what happened is that when he got a girlfriend, the girlfriend told him that he will have to love him with her kids, so they are his responsibility and everything that happens with them he sees them because they stay together, so with his own biological not that he is not taking care of them he does but I believe not like the ones he stays with.

INTERVIEWER: What type of support would you need to assist you in fulfilling your role as a father? [Probe social services, health care, livelihoods, self-image]

RESPONDENT05: As you have seen just now, as fathers we do not support each other, you cannot judge a father about what he does, what I need from you as my brother that I hang up with I need support and listening to me when I come to you with a problem not to judge me.

RESPONDENT05: as fathers, we have this mentality that being a father means giving money in the house only, and giving money in the house does not mean anything. We as black people that is why you find out that our kids end up doing wrong things or associating themselves with bad people because what that child knows is being given money and not guided about life. So, what I think the kind of support that we need is having leadership conversations and workshops where we will have

fathers who will groom young men to be good fathers in society because money is nothing without *ubuntu,* you will go nowhere with your money.

RESPONDENT10: we need support in terms of laws when speaking about children, in a case where parents are no longer together the law most of the time gives a mother more power over the children than a man. Now that disrupts the bondage between the father and his kids. So, we need support that is not taken from the constitution. We need the constitution that will not think that punishing a child is abuse. We need a constitution that will give us certain control over our kids. So, I think we need that support more than anything.

RESPONDENT02: what we need as fathers is respect, times have changed, in the olden days, you wouldn't find kids holding boys in the street in daylight in front of the elders now, it's a norm and there is nothing wrong in society when kids do that, kids have rights, if I beat a child, they can arrest me. Even at home if you are not working then the woman takes you for granted.

INTERVIEWER: What has helped and/or hindered you in fulfilling these role (s)? What hinders you from being physically, emotionally, financially present for your children?

RESPONDENT05: I think this is simple, the one thing that hinders a man to be present in the life of the kids it's the mother of the child, women take out their revenge by using the kids to pay back on us.

RESPONDENT07: what is hindering me from being there financially is that I am unemployed, but emotionally and physically I am there and if I do work, I do support my kids.

RESPONDENT 04: what hinders me is the mother of the child.

INTERVIEWER: What are the benefits of being an involved father.

RESPONDENT12: you see my sister, in marriage, I have 4 kids, and others outside of marriage and now there is one kid of mine who makes mistakes and do not want to listen to me, I do not want to lie to you my sister, I do not like that child, because of his behaviour, I am not happy with him.

RESPONDENT08: some of the things I like are that when I do something for my kids, they become happy, I like that feeling they appreciate me.

RESPONDENT05: okay, with my kids they are my best friends, and having to come back to them every day makes me happy.

INTERVIEWER: What are the challenges of being a father?

RESPONDENT 12: That problem I have just mentioned, of being a father and the child does not want to be corrected. That is the same problem I have.

RESPONDENT: you see, we raise our children trying to feed them and clothe them and giving them the best education, but there is nothing painful as seeing your kids not listening and doing right in front of you. Then the child responds negatively to what you are trying to do.

RESPONDENT P10: The challenge that we come across as fathers is that once you cannot control yourself as a father you are instilling a problem in your kids without knowing that, why am I saying that?

RESPONDENT 7: the challenges that I have seen as a father is that what I have noticed is that in the most black community as the boy child your father is responsible for you, like when I grew up, I did not grow up with my father and now my challenge is that when the boy child grow up most of the time

they look up from their fathers, so now my biggest challenge is that my boy is doing everything that I used to do I started drinking at a young age, and my boy also has started drinking, so now he comes back home drunk and late in most cases and he is only 17, he is stubborn and he sleeps in the backroom and so now when he comes back late and in the early hours of the morning I call him and speak to him about his actions. That is the problem I have right now.

INTERVIEWER: What do you find most difficult in all this?

RESPONDENT:7 what I find difficult is that he is stubborn but when he is sober, he listens.

INTERVIEWER: Is there anything you wished you would have done differently as the father?

RESPONDENT: 05 I wish that I had a baby with someone else, not with the baby mother I have.

INTERVIEWER: So, you are saying you wished you had a baby with a different person?

RESPONDENT 05: I wish that I could have children with one person because now we are separate from the mother it is obvious that we will not get married, I will marry someone else.

RESPONDENT 09: You know in the relationship things are not always smooth, so back in the days I would beat my wife in front of the kids, sometimes you find that you are angry from the things that have happened in the *shebeen* when you come back home and your wife will also add to that anger not noticing that you are already angry then I will respond by smacking her in front of the kids and the bad things that I have done in front of them it seems like they have copied all of that, they are now doing it. I have also been involved in drugs but a long time ago I think it was 1987/88, it was even before I even had them, and I stopped and continued with dagga only, so it looks like this is the trend at home.

INTERVIEWER: As fathers, how do you take care of yourself? [Probe physically, emotionally, economically]

RESPNDENT05: in everything that I do, I think of my boy, because the consequences will affect him, every action done by me, and it will affect him also. Typical example as men if we are friends, we sometimes are not truthful to each other.

RESPONDENT 07: there is a change, once you become a father, because whatever that you do you think about the consequences, and what the child will learn from that. I think the baby also makes you have a positive mind because you think differently.

RESPONDENT09: I think being a father is a good thing and I am happy that I am a father, as a father you have the power to change your children, for the better, no parents want to see their kids growing up to become nothing. Your kids are your treasure, I still wish that my kids become what I want them to become, I want them to become something big and go to school. I wish I can one day see them on TV. Because they have become my hope that what I did not become they will become that.

INTERVIEWER: what encourages you or discourages you to accompany your partner or your child to the clinic?

RESPONDENT08: If these clinics can open hours which suit us, like after 5 and weekends then as fathers we would probably show up.

RESPONDENT05: So, they have mentioned what encourages, on the other hand, what discourages on the other side is the society, it discourages us from having the support to our women and children. For

instance, if I may ask all of you here how many of you buy pads for your girlfriend, and do you even know the brands? Because if I walk with pads in the street and people will say I am not a man enough *(ISINAYI*) he is carrying pads, so those are the things that discourage us.

RESPONDENT10: The other thing that affects us as men is that we like committing to the standards of the society.

INTERVIEWER: If you have ever accompanied your partner to pregnancy care or taken a child to a health care facility, what was the experience like for you? How did you feel in the space? How did the healthcare providers treat you as the father? From your experience, what would you like to have been different? What can be done to support fathers who accompany their partners and/or children to health facilities?

RESPONDENT05; So, my experience was good, because other older people in the clinic helped me when he was crying to keep him quiet.

INTERVIEWER: **So, the staff, how did they treat you?**
RESPONDENT05: Madness, those nurses are crazy. For instance, they asked me why are you bringing the child, where is the mother? I'm thinking I am also his parent, whether she is here or not I'm here as the father, why there is that thing of questioning a man when they bring their kids to the clinic. That is why I am saying it's madness.

RESPONDENT 09: When you walk with your child on the streets or in the shop or taking them to the clinic it's a good thing and you can see neighbours and people cheering you up for doing that and that support is good. Nurses tend to make mean comments, why they ask us about the mother of the child when I bring the child to the clinic?

RESPONDENT13: I became a father at a very early stage I was not mature enough and, in those days, when you take the child to the clinic with the mother was the best thing, even though there were long lines, I was happy, I used to even change the nappy of the child and I treated them well. When even left for work she knew I can stay with the child, and she will come back find the baby okay.

INTERVIEWER: What can be done to support fathers in seeking health care services for their health?

RESPONDENT 05: I think that all clinics need to have a men's clinic, I think every clinic should have that department because when you go to the normal clinics and there you are with women sitting next to you and they just come and ask you in front of everyone, they ask you "sorry *baba* how can we help you"? obvious I will not be honest for instance if I have a problem with my private part, I will not say out loud that I have a problem with it, so if the departments in the clinic can be separated so that we can feel comfortable to tell the nurse why I am there.

RESPONDENT 10: I think it goes back to say, we still need to develop a healthy community throughout South Africa, where when we are sitting and thinking about the clinic, we are encouraging each other.

INTERVIEWER: **What messages (for example on posters or on leaflets that pregnant women could give to their partners) do you think would work to encourage men to come in and be involved in these health services?**

RESPONDENT 04: I would say that as gents we do not have enough information, for instance in the clinic there are posters displayed they do not even know what they mean, in my experience, let's say I impregnate someone after that the girlfriend maybe say can you please come with me to the clinic,

then I get to understand what happens with someone pregnant then I can explain to someone that if you girlfriend is pregnant do not run away from her because this is what she is experiencing and at the end of the day it's your child.

RESPONDENT: Pictures, I believe that you can understand something well if it’s a picture and written in big words, even if you are not educated as a person pictures display a clear message of what is being said.

**Project Name:**	Fatherhood & MNCH
**Site:**	
**Record Number:**	FGD_03_110621
**Transcribed by:**	
**Used language(s):**	
**Interviewed date:**	11/06/21
**Transcription date:**	
**Duration of the interview:**	
**Checked by:**	
**Checked date:**	
**Edited By:**	

INTERVIEWER: As we are speaking about fatherhood, what does it mean to be a father to you?

RESPONDENT: P1, it means taking care of your kids, making sure that they have everything that they need for them to survive, even if it means you buy groceries for them, clothes at any time, that is being a father.

INTERVIEWER: So, being there for the child buying things that the child needs….

RESPONDENT: P2, it's to be responsible. All in all, it's to be responsible and not running away from being a father. Being there for everything that your child needs, if they say your child is sick, you might as well not go to work but take your child to the doctor. If they say there is a meeting at the school of your child, you need to attend that meeting and go to work later, at school they will write you a letter to take to your workplace. That shows being responsible.

RESPONDENT: P6, being a father means being there for your child. To make sure that your family's needs are fulfilled. Like you can buy clothes for your children and that they do have food in the house.

INTERVIEWER: Okay, so what I am getting is that being a father is being present for your family? What are others saying?

RESPONDENT: P1, like they have said, being a father is being there for your child and also being responsible but also knowing your child, you must know your child as a father.

RESPONDENT: P4, can I say something? What I can say it's all about being committed. What do I mean about being committed? Being committed, in such a way that you must know your children. What I understand is that children need both parents, sometimes the child may need a mother and there is a time that your child may need the father. What I am saying is that you must know your child as a father in order for you to be able to

In short I am saying the child needs support full time. What I can tell you is that you can buy your groceries, I am not opposing anything but as long as you are not there to give love and show love to your children because love is important to your children. Because number one, the child will not get love from outside if they don't get it at home, number two, charity begins at home, when I say charity I mean I am talking about respect.

INTERVIEWER: How do you think fatherhood has changed in South Africa overtime? Looking you how you were brought up and looking now as your child growing how has it changed?

RESPONDENT: P4. What I can say is that it has changed. I will tell you the truth, most of us we grew up without knowing our fathers. Most of our fathers they were ignorant and others didn't face the truth. Sometimes as men we have a wrong mentality, we have this thing of saying we don't care, you are a woman and I am a man, forgetting that these things hurt the child and by doing that when the child grows up will need both parents. Why do I say it has changed? Most of us because we grew up without knowing our fathers even though the mothers are denying us accessing to our children, you need to continue fight to see your child, when you get there you have that orange or chocolate or money, even though the mother doesn't want you but because you know how painful it is to grow up without a father. That is why I am saying it has changed, many guys want to be part of their children's lives. There are very few guys who don't want to be part of their children's lives. All of us as guys you will hear them speaking about they need to do this and that for their children.

RESPONDENT: P3, as the guys are saying most of us are raised by our mothers, most of the generation here were raised by their mothers and that thing hurt us a lot that is why we have so much anger and we end up shooting and stabbing each other. Now things are easy because we can talk to our kids and reprimand them. If you look now our children love us more than they love their mothers. Even though we didn't get that love from our fathers it's okay but we keep telling ourselves that our kids won't grow where we grew up because we know how it feels. Imagine all the time people are asking you where is your dad and at that time you don't know where your father is but your father is alive and doesn't care about you, so we make sure that we are always there for our children and it doesn't matter what happened but you know that the child is yours.

RESPONDENT: P2, I don't know my father. What I do is that if my child says I want an All-Star, I take my child and buy that shoe that the child wants.

INTERVIEWER: Okay, the next question is. If you chose to have a child, why did you choose to have a child?

RESPONDENT: P3, sometimes you don't choose. It just happens. You will find that you are busy saying to this lady you like her and then it happens that you get along together and God sees that these two want this and then He gives you a child and the lady becomes pregnant. Some become pregnant by mistake, you didn't plan for her to get pregnant and that becomes a blessing.

INTERVIEWER: let's look at the firstborn child, when you had the child did you choose to have the child?

RESPONDENT: P2, no.

RESPONDENT: P3, we were just playing.

RESPONDENT: P2, I Have four kids with different mothers. At that time I wasn't ready for the child but they came.

RESPONDENT: P1, okay, we planned to have the child, we wanted to have the child and then the second born wasn't planned.

INTERVIEWER: Okay, P1, like you said you planned to have the child, when you found out that your partner is pregnant, how did you feel when you found out?

RESPONDENT: P1, I was excited. The excitement I had was I was always making sure that I am always there, I would make sure that my child doesn't lack anything.

RESPONDENT: P4, I was overwhelmed. You see when you plan for something and it doesn't come true but when you plan and it comes through that thing is life-changing, so I was excited because I planned it, I wanted it and I got it and I asked for it to come.

RESPONDENT: P5, I will speak about my story. I planned to have children, I have been with my partner for 18 years, we got married before we had children, we planned our first child and I told myself that I want to have children because I love children and then after she got pregnant that is where she found out that she is HIV positive. So, we had that child and we planned to have children. I couldn't go out and have other children because I am married to my partner. I had wished to have more children but my situation became difficult because of our HIV statuses but I would have loved to have more children.

RESPONDENT: P5, yoh! I was excited. I was excited because when she became pregnant I had told myself that this is the person I want to spend the rest of my life with and that is why I married her even before we could have kids. I was excited.

INTERVIEWER: Okay, as a man knowing that you are going to have a child, how does that change you?

RESPONDENT: P5, I feel great. I become happy when they call me like that. Since the child came you know they say that action speaks louder than words so I became very happy.

RESPONDENT: P6, you know when you have a child and in the community, they call you by saying "Zinhle's" Dad that gives you so much respect in the community. It gives you that respect of saying that you are a father. Like for example if you don't have a child and you only have a wife they don't take you that seriously but if you have a child they take you seriously.

RESPONDENT: P7, I can say, when you are a father and your child has friends and when you reprimand you won't reprimand your child only but everyone, so being a father gives you that respect.

INTERVIEWER: So we want you to tell us about your life experiences and the relationships that have had an impact on you and your child whether you are present or absent in your child's life?

RESPONDENT: P4, like I said earlier sometimes our women will deny us our children. I have four children with different women but the last one was already there when I started dating the mother, I raised the child since the child was 1 year 6 months until now, the child knows me as the father. I don't participate a lot in the child's life because I am not the biological father because sometimes they want the biological parent that is why I don't participate a lot in the child's life but the child thinks I am the father. It hurts as I mentioned that we want to be there for our kids but despite the situation whether I am working or not, just to be there for your child. You know I once experienced a situation that sometimes people judge you on the outside and they are done. It happened that at the ladies' place they didn't want me and when the lady had the child, the family made sure that I don't see my child. Now you see what they are doing, they want to chase me away from my child and I know very well that child is mine. They don't want me to see my child but I want to see my child with my whole heart. That experience makes me give up on my child. If I go there without their permission they will take me to jail and I don't want that I just want to see my child. Secondly, this thing hurts this way, I won't be able to support my child, ended up staying seven years without seeing my child. You are lucky if at the girlfriend's place they like you. Most especially if you are employed, they see something that can be used especially the in-laws. When they see you, they see someone they can ask for money for

cigarettes, alcohol, and all those things. So, you have to abide by that because you know what you want there and you have to abide by their rules but most of the time, this thing of seeing our children, as I said earlier most of us grew up without our fathers and that thing is painful, that is why we must always be there. Brothers, we must always be there for our children. It doesn't end in making children.

INTERVIEWER: What is your role or roles that you are playing in your children's lives?

RESPONDENT: P7. I think as a parent the most important thing is to look back at the way you grew up and try to bridge that gap that was there while you were still growing up, if ever while you were growing up your father or mother were not there, you just try and close the gap of being a parent that is there.

RESPONDENT P9: First thing first is that as fathers we need to talk to our children and let them know what is happening. We need to let our children know, I think as parents we are failing our children because we are not being transparent to our children. We need to teach our children responsibility and let them know that when they grow up they will go through these things.

RESPONDENT: P10, what I could say on my side is that the role that I am playing in my children's life is not only financially but I am physically there as well. Even their mother would complain when I get home from work because my kids want to be next to me and it will be like I am shutting her out. You get to hear what other people are saying and you get to see where you are going wrong as a man because we always point figures and not fixing. Now I am present physically and emotionally. I am always there for them and I ask them questions so that they know that once they start to do something they know that their father will pick it up very quickly because I am there. I explained to her things that have happened between me and the mother and she has seen how it affected her. I am back and I want to fix things with them. Because it's painful as a parent if you hear things from the community and you are not there. It's very painful to hear from people that since you don't stay with your children this is what is happening. I had to look back and see what I had contributed to the situation for it to be like this. Most of the time we fail to identify the problem within us and we try and find the problem in other people. I

RESPONDENT: P5, We must treat our children the same. If you have three children you must treat all of them the same. When I was still a kid I had four siblings and we were not treated the same. You see how old I am right now but that thing is still there. I have that thing of why did this happen to me and this thing is done by parents and its hurtful thing but they don't see that this thing is hurtful and it was seen by us as children and I don't want my children to go through the same thing that I went through because it will hurt them in a big way.

INTERVIEWER: What are the roles that you are playing in your children's lives, how do you manage to fulfil those roles?

INTERVIEWER: Like you said all three are important, being physically there, emotionally there as a father, the question here is what kind of support do you need to be able to fulfil those roles as a father?

RESPONDENT: P1, I would say employment, time to do all those things. That is the support I need to be able to do all those things. If I can get employment and then I can support my child and if I can have time, because you do watch movies and you see all those things being done, they go and watch soccer and all those, I think that is the kind of support I need to be able to do all those things.

RESPONDENT: P3, Money, and Love. If I know that I am being loved and I have money, that is all the support I need.

RESPONDENT P6: Okay, for me, I would think it's finance and appreciation that is the support I need. Because its hurtful that you do things and at the end of the day you don't get appreciation from that person because that support will make you do more even tomorrow. Obvious finance makes things easy to support them. If there is sugar in the house then everyone is happy. Because I have a five-year-old and whenever she sees KFC advert will make comments about it and then tomorrow you go and buy that it makes the child happy, even with my partner when she sees the KFC advert and comments that they wish we can get it and then you get it and they know that they don't cook but if there is no sugar in the house it makes you feel down. When a person appreciates you it says that you did something that they are happy about.

INTERVIEWER: what is your best memory of your child?

RESPONDENT: P2, when it's the child's birthday and even when the mother is there and we go out together. Even when it's during the December holidays and I get to be with my child and then when we go and buy clothes the child chooses whatever he or she wants. If the child says I want Nike the child gets it, I go there prepared.

RESPONDENT: P4, My best moments, in all my children when they were all born. It doesn't matter which mother. I was so excited it's like the heavens were opening, those are my best moments. My other best moment is when I am talking to my child. Because that allows me to see that I can bring someone into the world and I can talk to the child and have different opinions. My children are all grown up, so the best moment for me is to have a conversation with my children, more especially when they are still young and they are not able to talk properly I love them.

INTERVIEWER: The question here is what are the rewards of being a father?

RESPONDENT: P4: For me the most rewarding is that taking the child to school and seeing her report that she has passed at school and she got a medal, ever since she started going to school she has never failed. That feeling is amazing. That's the reward for me as a father.

RESPONDENT: P3 - The child came back from school with a star on his forehead and he was happy and if he didn't do his homework he cries and tells me that they are going to reprimand him at school. So, that shows that I am raising him well. That is my reward.

INTERVIEWER: Okay, as much as there are rewards but there are challenges as well. As a father what do you find most difficult being a father?

RESPONDENT: P4- Being away from my kids that is what I find to be most difficult. Like, I will tell you even the one who is in Swaziland. He knows that he looks like me and I am his father. All of my children know that I never denied them when their mothers tell me that they are coming.

RESPONDENT: P4, When a child passes away is something that is most difficult, I have never experienced that but what I can say about the most difficult thing about being a father is when your child doesn't listen to you.

RESPONDENT: P1, You see a challenge that I had with the partner who tells your child your problems and then wants to use the child against you or you buy things for the child and the mother doesn't give those clothes to the child just to spite you. That also becomes a challenge. Even the parents of the lady will be against you, just like my brother said earlier that they judge you the first time they see you, that is also a problem.

INTERVIEWER: Is the anyone who wants to add on what they could have done differently as a father?

RESPONDENT: P1, what I could have done differently as a father is that getting a partner who wants the same things as I am. Must have children with someone who wants the same thing as me and not just going around and having kids all over the place, I think I would have family right now that I am staying with right now.

INTERVIEWER: Which legacy do you want to leave your children with?

RESPONDENT: P1, I love to leave them with a business. Even though it opens and closes but I want it to be run by the one when I am no longer there.

RESPONDENT: P4, Legacy is what you leave behind according to me. It's something you planted. The beginning of wisdom is to do something that will benefit those you are leaving behind. In short, we need to plant good deeds in our children so that they can be rooted, that will make them stand strong and face the situation.

INTERVIEWER: How do you take care of yourself physically, emotionally, or financially? How do you take care of yourselves?

RESPONDENT: P4- As an HIV-positive person, I need to make sure that I take care of my health and also think about the child I will be leaving behind if I don't take care of my health. I shouldn't be careless with my life. You are sending a bad picture to your child when you don't take care of yourself.

RESPONDENT: P1- physically….. eish I don't like to train but I am very choosy when it comes to the food that I eat and then emotionally I always try and make myself happy, I will go out with my friends and hang out with my friends and I love watching sport. That is how I take care of myself.

RESPONDENT: P5, I would like to comment on the emotional one. I wish I could be able to handle my problems when I have problems as a man of the house and also to be able to get good advice, as we are advising each other. My wish is that I can be able to handle my issues. As we are speaking right now I do get it and it's staying inside me because I sometimes struggle with handling issues in my family.

INTERVIEWER: Okay, thank you for that. Has becoming a father changed how you look at yourself?

RESPONDENT: P4, I like this discussion. I would have died if I didn't have kids because I didn't have a reason to leave. I was doing things the way I want to. So, being a father has made me be committed to my life. I have a reason for living now. Now my kids are a reason for me to live, I live to support my kids and be committed to them. I live to support my kids that is what makes me live, I live to be there for my kids, if I didn't have kids I don't think I would still be alive even now.

RESPONDENT: P5, as my brother is saying, when I think about my kids I always think of if I die how would I leave my kids behind that is why I always come for my appointment dates, I try and make sure that I live longer so that I can see my kids growing up.

INTERVIEWER: What do you think can be done to make the experience for the fathers who bring their children to the clinic, how can we improve their experience?

RESPONDENT: I can speak on my side. I can say encouraging us, talking to us. As you are a nurse when a father brings the child encourage him and talk to him, have positive words for the father so that he gets encouraged.

RESPONDENT: 4-, I think if we could have programs such as Male corners they are needed, like at Itereleng they have that, while you are waiting there, someone comes to teach. Because you come to the clinic and you are sitting there and doing nothing I think that is what makes men not want to come to the clinic. I think having that will keep men engaged and it will encourage them to come to the clinic even more.

RESPONDENT: P6- recommendations we are getting from the nurses because there are fewer guys who are taking their kids or accompanying their partners to the clinic. So, it becomes a surprise to the nurses when they see you bringing the child, like my brother said that they ask you questions like where is the mother if you are the one bringing the child to the clinic.

INTERVIEWER: Okay, maybe when you get here to the clinic what kind of service would you like to get or see when you are here at the facility?

RESPONDENT: P4, it's simple I think respect goes a long way. You will find that people who hold higher positions have a problem with respect and they need to be trained professionally about whatever issues they have they shouldn't bring to work. Because they need to leave their problems at home because the people they are dealing with have their pains so, it will be very harsh to that person.

RESPONDENT: P2- It is sad when we come to the clinic and then you get disrespected by a woman that thing is not nice. Women can bear with it cause you can talk. This thing ends up making us not want to come to the clinic because you will think of the disrespect that you will get from the nurses but when you look at the doctors they respect a patient a lot but the nurses are disrespectful.

INTERVIEWER: Can you tell me what will be an acceptable incentive for fathers who are accompanying their partners to the clinic, what can we do for fathers?

RESPONDENT: P3- What is needed is something that is written and that will be something that is used over and over again. Maybe it can be a cup that is used to drink coffee or glass or the flask that you have just shown us. If you look at the cup or plate that is being used every day that has information but if you give me this paper I will look at it and throw it away but with a cup or plate I will see it every day and it will remind me of the benefits that are found at the clinic. what I can say it should be physical but not something big.

RESPONDENT: P5, The suggestion that my brother has made is good maybe a cup or plate that will be good. Can I go back a bit? Going back to talking about experiences back in the rural areas there was a situation like that where the nurses and the people want to be respected, you see when they say go to this room and you go and after a couple of times, there was a suggestion box and ever since that suggestion box was put there things have changed because when I go to this room and I see that it's this nurse and I see her name and when I leave I have a comment and put in the box. They were lots of problems but after having that suggestion box put in place made a huge difference in the clinic because you can write a comment and no one will know what you said.

INTERVIEWER: What messages for example on posters or pamphlets what can be given to pregnant women to give to their partners to invite them to come to the clinic? what would you like to see written on the pamphlet to invite a man to come to the clinic?

RESPONDENT: P1- I would make an example at the ANC, when a child gets there, he or she gets a t-shirt written "thank you for coming to this clinic, thank you for being the father of the day" I think that would motivate them because you would see that every time and you would brag about.

RESPONDENT: P2, like most fathers, love soccer, so if in the pamphlet you can have something around that, I think that would motivate them and make them interested in coming to the clinic.

**Project Name:**	Fatherhood & MNCH
**Site:**	
**Record Number:**	FGD_01_180521
**Transcribed by:**	
**Used language(s):**	
**Interviewed date:**	18/05/21
**Transcription date:**	
**Duration of the interview:**	
**Checked by:**	
**Checked date:**	
**Edited By:**	

INTERVIEWER: What does fatherhood mean to you?

RESPONDENT: P04, to me being a father means that I have a responsibility since I have a child that I need to take care of to making sure that the child is taken care of in all the sectors that the child might need. And also, be supportive in everything that is happening in a child's life.

RESPONDENT: P06. Being a father means being there for your child in every way not only financially, but you have to play your role because as fathers when we give money to our children, we think we are there for our children.

RESPONDENT: P02. It's to be an example to your child, like for example some of us did not know our fathers so in the future we end up becoming fathers who do not care about their children because our fathers were not there for us.

RESPONDENT: P08. To be a father means to be there for all your children even those who are not biologically yours, you have to be there because a lady can have a child and I start dating her while she has that child, I have to be there for the child especially a girl child.

RESPONDENT: P10. I hear what my brother over there is saying. Having a child is not your choice, you can have a boy child or a girl child, having a child is a blessing. In your family, you can all be boys and when you have a child you have a girl child. It doesn't matter if you have a boy or girl, the only thing is that a child is a blessing no matter which way the child came, even if they say in your family there are boys only and you come with a girl child, be there for that child, no matter what kind of situation you come across you must continue to raise your child because if you don't, they will end up being raised by step fathers and it will look like the step father is the true father to the child even though you are there.

RESPONDENT: P05. Being a father is to be there for your child from a young age, teach the child how to communicate with people, to be free, being able to express, like to instil confidence in the child to see examples in you and to see a friend in you until the child grows up, so that you don't become a stranger when the child sees you, that is what I can say about being a father.

<u>RESPONDENT</u>: P01. According to me being a father, I will call it a maturity level in life. I call it a maturity level in life because of the picture you are playing in the child's life. You can be an uncle to the child but play a father's role in the child's life, you are there for the child physically, you are there for the child emotionally, you are there for the child financially. You can play those roles in the child's life until they grow up to what they want to be. I will make an example. It might happen that we are all staying in the same house all of us as children but there will be that one person who will play the role of the parent in the house and be there for the children in the house, as I am saying it's the level of maturity in your mind, you can be young physically but if you can take care of that child-like holistic, you are playing a father's role in the child's life. We can be from the same township, and I can play a father's role in the children from my township on the things I engage them on so that they can grow up and become what they wish to become in life.

<u>RESPONDENT</u>: P03. To be a father you are not a father to your children only but to the children of the whole community as P1 has said because what I teach my child, I am building a mirror in that child, whatever that child does, that child is representing me as a father, if he or she swears then that shows what kind of a father I am. I am not supporting the child only. Even when the mother is pregnant, I need to be there from the beginning even when the child is born, I need to be there, that is how I see it.

<u>RESPONDENT</u>: P11. To be a father to me means it starts being a man by taking responsibility not only where you stay but also in the community where you live, by taking responsibility, you have a responsibility as a father, if there is something wrong that the child has done even though that child is not yours, you have a responsibility of showing that child the correct way of doing things.

<u>INTERVIEWER</u>: **Okay, thank you all for your opinions, with all your opinions about fathers it leads to the next question about fatherhood. How has Fatherhood changed in South Africa over time? In your own opinion, how do you see fatherhood has changed in South Africa?**

<u>RESPONDENT</u>: P02. When I was growing up my father was driving trucks so most of the time he was not around, as he would be gone for about 2 to 3 months and when he comes back he brought bags of chips and when I grew up I saw the impact of that when at school they wanted my father, my uncle will go in his space because my father is not there and it will look like I don't have a father whereas my friends knew my father but he is working that is understandable. So, I didn't stay with my father. So, coming to me, I have played soccer with my kids, chasing around the house, I take my kids to the stadium, my daughter is still young but I do play with her in the house, I sit down with her and have quality time with her. So, that has built a good relationship with my children because of making time for them. Even when you speak with your kids don't be harsh or loud to them rather speak to them at the same level when speaking to the child.

<u>RESPONDENT</u>: P12. I think the educational level of our parents was not there. The system that was operating at that time made it hard for them to be there for us and then we adopted that, I will speak about myself. I copied it from my father. My father was not there but he was there and then I did that with my first child. Fortunately, I had the opportunity of going to school, the second child is 18 years, she is my friend we share about everything. So, there was a transference from my father to me and then from me to my firstborn. I think education played a role and the system of the country played a role, the environment, the TV, the radio and you do copy from the movies when you see a father and his child.

<u>RESPONDENT</u>: P03.to add on the point that has been by my brother. In the old days whatever you do as a child you were told that you are a child but nowadays a child is told that he or she mustn't listen

to anyone, forgetting that as a person you grow with the help of another person. As a person, you need the help of another person. In the old days, we were told that we need to go and play with other kids and when you eat, you eat with other kids but now a parent can give money to the child and told not to share with other kids. That thing builds respect amongst children.

RESPONDENT: P04. fatherhood has changed in the generation that we leave in. Men are stepping up and they are taking responsibility to be there for their children. You will find that it is us men who are fighting to be there for our children unlike before, because you will find that as an absent father the child will grow up by saying "when my father was still around" I will make an example with me, I once had a problem where I ended up leaving the mother of my children, I was doing everything for them. Me not being there for them that year I sort of realized that the community was even starting to complain, children are always dirty, children stay out until late. And then I came back and told their mother that we are fixing this and whatever has happened between us, it shouldn't affect the children. So, what I have seen is that most men are fighting to be part of their children it's just that the mothers don't want them to be present.

INTERVIEWER: **Your experiences and what you have seen that have impacted your fathering.**

RESPONDENT: P02, Okay I will start at home. My father was a truck driver, and he was drinking, and he will come home at night drunk and then they would fight with my mother. They would fight in our presence. So that thing will have that thing because I can see it now that I sometimes will have anger in the house and then fast forward to the year 1994 and 1995, I wasn't working around that time, my sister bought a house in Delmas, when my sister's kids where at school and other children were giving them a hard time so I had to go and stay with them so that they would find someone when they come back from school, so the love I saw in that couple, I always tell them that they are my role models, even when they fight the husband will leave and sleep in the car, even the way they treat their kids, they are treating them in a right way. I have given you two scenarios and I had to choose which way do I want to take.

RESPONDENT: P12. I have got a very similar situation. I was raised by a father who was staying everywhere, and I was raised by a shebeen queen and I'm very grateful for that. She wanted nothing but the best for her children and on the other hand, I have a problematic father, he had girlfriends not far but nearer. My mother will always say we won't be like our father, she would say positive things and these things built me but when I grew up, I ended following in my father's footsteps and ended up being a player, falling in the same trap that my father fell in, by God's grace I have the agape kind of love. The kind of love that I have inside of me has risen and I was able to go to school. In terms of schooling, I have a bit of background in terms of psychology, on how to treat people. And then my understanding is that the things that I was taught by that lady back then, are the correct things and the things that you adopt in the environment are the wrong things. Then there are stages that you go through such as adolescence and puberty, to get out of the stages is kind of difficult.

RESPONDENT: P01. My experience was kind of big. The first person to show me the role of playing the father figure role was my grandfather. He was in a polygamous marriage. And it was kind of difficult because my other grandmother was Shona and the other one was Ndebele but the way he was able to mold them like no one would separate his wife. Yes, at home there would be problems and my mother knew that during school holidays I don't want anything, I just want money to go to my grandparent's place, I know that it was the most conducive environment where I could do anything I want but I would also be called into order. The other role model is my younger brother, he is the youngest amongst us all, but he is the one who is guiding us, but we should be guiding him. The other thing where you could see that the father figure is missing is when you go to the orphanages or

creches. You will find that they stay with mistresses, and they are there but when you are men and you come in there and you take one child in two minutes that child is fast asleep, that shows that warmth that is what is missing. That is where I saw the importance of playing the father role figure.

<u>INTERVIEWER</u>: The next question is we are looking at roles, what are the roles you are playing in your children's lives?

<u>RESPONDENT</u>: P10. For me, everything I do is for my child. Even though the first time I denied the child and then I was allowed to have the child but everything I do for my child. Even when the mother of the child would say "you are only doing for the child" and I would tell her that this is my child, and I would do what I see is best for my child because I don't want to deny my child the opportunities that I didn't have.

<u>RESPONDENT</u>: P04. On my side, I have accepted that I have three kids. The first born I am not doing anything for that child, the reason being the mother of the child. She took the child out of South Africa and took child to Swaziland while the child was still young. When I ask her to give me the numbers of the person, I can get hold of that I side to speak to my child, she refuses, he wants us to get back together, and I do want her. With the other two children, I have been there for them, I do everything for them.

<u>RESPONDENT</u>: P05. My first born is 6 years old and I am not playing any financial role but that is my first-born son, I have fought to have communication with him, even though he stays with his mother and his mother is married, financially they are fine. What made me step back financially from him is that you know when you buy him something and when he comes back, he has the same thing but a better version, so I chose that I would be there, advising and showing him that I am there for him as his father. And then, the two girls I see them every week because they do come and visit and we talk, they know that their father is there and then I have two nieces, their mother who's my sister passed away in 2013 they are close to me than my kids because we stay in the same home. I have taught them most of the things, I have taught them how to cook if they don't understand something they come and knock at my door and ask me. Sundays they know I love watching Mzansi Magic, they come and watch tv with me and they sit with me not their grandmother and we watch tv together. With them I'm closer than my kids but I do support my children.

<u>RESPONDENT</u>: P07. I do support my children some times. My father never gave me any support, he was a soldier. I would see him sometimes and then for a long time he is not there, my mother was busy with other things. I was raised by my grandmother, I never got anything from my mother. Even though now I am staying with them I try to be there for them.

<u>INTERVIEWER</u>: Thank you very much gentlemen, the next question is to what extent do you think you are managing to fulfil the role of a father?

<u>RESPONDENT</u>: P06. I can say our communication is good because I communicate with all of them. Even the one who is 6 years old, prefers to communicate with me more than his mother and we do everything together. Everything is going well.

<u>RESPONDENT</u>: P04. On my side, I can say I failed on those who were close to me for two years, but I have fixed things with them but with the one in Swazi it's still difficult.

<u>RESPONDENT</u>: P05. What I would say is that communication is there and everything but what is lacking is that the boy plays soccer and I have never seen or gone to the game to watch him play. With the girls, the second born is doing well at school and she was nominated for the top achiever in school but just being there and see, I think I lack that visibility when they achieve something. I think when you do

something as a child and seeing your parent there it somehow motivates you. I can say that is where I am lacking but am trying to find a way of fixing that so that I can be involved in the things that they do.

RESPONDENT: P11. In terms of communication with my children it is there especially with the one I am staying with because I am married to the mother. The others stay in Eastern Cape with my parents. The challenge that I have is with the child that is not biologically mine. Even if the child doesn't want me the child must be fine. I even spoke to the mother about this child and told her that the child must know where he comes from, and they must communicate with the child.

INTERVIEWER: **The problem that is preventing you is the mother of the child?**

INTERVIEWER: **Those who have answered me the common factor is the mother of the child or maybe I can put it this way. To those that are not supporting their children, what are things that are preventing you from supporting your children?**

RESPONDENT: P01. What I have noted what makes us not support our children, remember when we started earlier, we spoke about having children when we were still young, and then came the issue of paying for damages. Where am I going to get the money for paying damages whilst I am still young, and I am still at school? I had a child in 1999 and I still don't have a stable job where I can say I can go and pay that R2000 for damages that they wanted. I still do things for the child but there is still that thing that I need to pay for the damages and the child is using the mother's surname. The child cannot change to mine until I have paid the damages. I can't include the child in the policies because the child is using the mother's surname. Because even now the person I am staying with is using a different surname. I think this issue of paying damages is a problem

RESPONDENT: P03. I would put this on the parents of the girl. The parents don't appreciate what you do for the child. It won't be enough; they would compare your support with the support they were getting in the past. No matter how hard you try, they would always compare.

INTERVIEWER: What is helping you to play your role as a father to the one you are playing a role in?

RESPONDENT: P06. What is making us not to be able to support our kids is because of the mothers. I wasn't working and I was gambling. I had my corner, I was able to buy my kid a hamper and my child would tell me that they are eating it at home when he is at school and when I try to address that with the mother, she would tell me that I should take my child. I would come back and give the child money and we would agree with the mother that I will pay the school fees and transport money and the mother must buy food, she would turn against me and say I must take my kid. So, I told my child that when you come back from school you can come to my house and eat there.

RESPONDENT: P12. I will focus on the firstborn child. My firstborn child is on *Nyaope* (drugs), so he steals so that he can get his smoking. If I can come to him and say here is money go and buy a trousers and a shirt, he will take that money and smoke it. When I ask him let's go and ask for help, he will run away and come back after three months, and then we have to start all over again. It's a big challenge for me. A relationship with my firstborn child doesn't exist at all. Plus, he is grown up, he is 28. The decision that he took to start this thing, it's the very same decision that he must take to get out of that situation.

RESPONDENT: P01. What I have seen is that as guys we make children all over the place. And you get 10000. The mother in Soweto is expecting 2000 from you, so if all the five mothers expect 2000 from you, you will end up with nothing. It will go back to the thing of prioritizing because the mother on this side is not that demanding, so you will push this side. So, this thing of having kids everywhere is killing

us. Because the expectation is not the same, if all the places are the same then we will end up hurting all these people, we won't be able to give them all the support that they need.

<u>INTERVIEWER</u>: Thank you so much gentlemen, is there any support you need, maybe you think you need as a father and those things make fathers not to be alright, maybe it can be social services or healthcare. What kind of support would you like to receive for you to be able to fulfil the role of a father?

<u>RESPONDENT</u> P01: Most fathers need a lot of engagement, why am I saying this? Because we are from broken societies you understand, through the way we grew up, we grew up in the environment where our mothers were beaten up and we thought its normal, we grew up in an environment whereby alcohol comes first and it's a norm, and it balances with the previous question that when you grow up with your father and you connect with your father emotionally and you become one and there is nothing that can separate you from one another. When you connect emotionally with your father, there is that stage where the mother is pregnant and sometimes the father has to brush the stomach and by the way, both of you are pregnant and you are connecting the three of you. Now, we did not have that, because the father was drinking somewhere while the mother was having pains at home. Automatically there is no bond. Remember what you learn is what you hear and what comes through your ear, he is drunk, and he shouts and swears at your mom and automatically we know that is a life that is how a man treats his family. So, we were broken from the beginning when we were kids, hence we are broken even now, hence breaking others, if we were raised with love, it will be easy to communicate our emotions to our partners to say, please love me, I do not have money because I am unemployed. Connection is important and if we had that we were not going to run away from our kids instead we were going to sit them down to say, my child here I am I have tried but here I am if there is anything you need, I will do it for you if I have the means, instead of running away and leaving them with a lot of questions. So, what I am trying to say is that as men we are broken and it's a cycle that continues forever.

<u>RESPONDENT</u> P12: to support P1 I think men need to be educated to understand child development phases, for instance, I will not be able to support my child with R500 who 5 years are old, that will never make sense. We need to spend time in their different phases of development, if we can understand that then they can come close to us, it is not only going to be financially but also emotionally and physically, so that we can understand them holistically, rather than giving them money only, then what after that? A child that is 12 years old has been given R10 000. For what? What does that child do with so much money, then you are turning that child into something else, that is no longer a child, what you are doing to the child you are making the child not be able to be self-sufficient. Therefore, we need to be educated as Fathers and understand all the stages.

<u>RESPONDENT</u> P07: Society sometimes expects more from a man, now that person looks like someone useless or unsupportive, now they do not count what has been happening in your life previously, things such as you were unemployed, and they expect money from you while you do not have.

<u>RESPONDENT</u> P12: our image as men are dented there is gender-based violence which is all over, the media perpetuate wrong things and bad things about men, the only beautiful things on the adverts are associated with women, you see the adverts women helping a child to walk and feeding them and all that you hardly see a father holding a child in the advert, the whole system in the media does not show fathers as good people, they shows fathers as monsters, nothing is beautiful that comes from

the social media about fathers, even the father's day, for example, you do not see the hype like the mother's day, so there are double standards from our society.

<u>**RESPONDENT**</u> P10: There is something that I would like to raise when my child was young maybe he was 3 months, and he needed to go to the clinic for vaccination and the mother couldn't make it on that day, the mother was telling me that she cannot make it, so I told her that I will take the baby to the clinic, when I got to the clinic I only found women only, I was like Yeer, so the nurses gave me that look, and asked me what I am here for, I told them I'm here to vaccinate the child and they asked me where is the mother of the child? I told them she is not here, so I did what I had to do and after that, we went to eat with my child.

<u>**INTERVIEWER**</u>**: Thank you, can I move forward, what are the best memories you have about your child?**

<u>**RESPONDENT**</u> P03: I think he was 3 or 4 years; I took him to East gate mall, so when we were there at Checkers hyper, he wanted a toy and I bought him the same toy he wanted and he always reminds me that do I still remember buying him that toy, even now he remembers that, and it makes me happy that he has good memories of me.

<u>**RESPONDENT**</u> P09: So, during Covid-19, my child was staying in Tembisa with her mom, for the whole year, so when the schools were opening up, they called me, I still remember it was very late when they called, so I spoke to her and she said she wants to come to stay with me, that was the best moment ever, so every day when I see her, I don't even know how to express it but she also sees my excitement of staying with me. so, leaving Tembisa to come to stay with me was the best moment ever.

<u>**RESPONDENT**</u> P04: In 2010 when President Mugabe came for the visit, I was a guard, so whenever there was someone big who is visiting, they will choose a guard that will look after that person, and fortunate enough they choose me. So, they said I must bring my family, I took all my kids there, everything was catered for, and when we were eating, she took the fork and knife and used it I was so happy to see her, I said to myself wow, my child can use a fork and the knife now, I was happy because it showed me that I have done some good work. That was a best moment.

<u>**RESPONDENT**</u> P01: One of the moments I will never forget in my life, when I just hold him for the first time when they come back from the hospital, just to hold him in my chest that picture remains forever in my mind. It's nice. Then the daughter, we sometimes speak with sign languages in the house so that others will not hear what we are saying. So, the other day, she came to me, and she said she wants to teach me sign language, she taught me how to say I love you, her narrative is that 'I'm one, 'love' is 4 and 'you' is 3 because of the letters that contain in each word, now that was priceless also.

<u>**INTERVIEWER**</u>**: What are the rewards of being a father?**

<u>**RESPONDENT**</u> P05: The best reward is seeing what you have invested in your child showing, seeing them passing at school, I think it would be sad for the father to help the child with the homework and when they come back from the school and tell you they have got 0 from the homework we did together, you understand? I think that will discourage the child, but if you do something for them and they are happy and you get their smile, those are the rewards for me.

<u>**RESPONDENT**</u> P07: with my kids, they are always calling me and telling me that they miss me and want to stay with me, the older one she will send me SMSs, then my second child I even get compliments

from people saying, “your kid is smart”. That makes me see that I am doing the right thing and I must continue to do right by them.

RESPONDENT P02: The reward that I get is that all my kids have been to school, and they can assist each other, and sometimes If I do not have money, they help me with transport money. So, they know each other, and they can assist each other.

RESPONDENT P09: The reward that I get is that I can be there for my kids emotionally, I never had that emotional connection with my dad I wanted to have that. So when I got my first child, I just wanted that connection, and I have maintained that, I always wanted to have that connection because I hold it dear to my heart. So, I feel like it’s a huge reward now because when I meet my firstborn on the street, she can buy me a cool drink because I have always been there emotionally even though she did not stay with me.

INTERVIEWER: Okay, the next question, what are the challenges of being a father?

RESPONDENT P05: I think one of the challenges of being a father is that everything is facing you or on your shoulders. You find out that you get a partner who will expect you to be a provider in all aspects, instead of meeting you halfway. I think that is most challenging because they have an expectation and now if you do not meet them that looks like you are a failure as a man.

RESPONDENT P02: I think what P5 has said is the common denominator, being a father has too many expectations and responsibilities attached to it by society what he is saying it's true that we will not be able to do everything it is happening.

RESPONDENT P10: The challenges that we come across is that once you start this journey of being a father, so sometimes you need to do time management, time for friends, and time to spend time with your kids, because sometimes there are people who do not have time for their kids, they will say ahhy we always see each other at home. But when you spend time with them you can see where they are lacking and where they are strong, and you can also be happy as a father because that is how you can determine if your kids need assistance in certain things.

RESPONDENT P12: I think what becomes a challenge is we become fathers while we are still young and now the most challenging part is that we are still in the adolescent stage and now we need to play a role that we do not know anything about, how do you direct the child into the right path, then the responsibility becomes too big then, and becomes a challenge.

INTERVIEWER: Is there anything you wish you could have done differently as a father whether in the past or now?

RESPONDENT P07: what I wish now is that I wish that I was there for my babies when they were born because when my kid was born, I was not there, so I only find out when he was grown up that he is mine. So, the connection between us is not the same as it would have been while he was young, you know how they are working and old and can do everything on their own without me.

RESPONDENT P10: You know when the mothers just finished from the labor ward and then she needs to go to do the birth certificate for the baby like the home affairs if I knew I would have taken both mine and hers, so that both appear in the baby certificate, because sometimes babies die, now maybe they will be asking if this child did not have a father, and if we were both in the certificate it would be clear that the child had both parents.

RESPONDENT P08: I wish that I did not deny my son back then but because I was young and scared, but as much as I denied him, I was always there for the mother.

RESPONDENT P05: what I wish is that I would have planned my babies, you know, having to plan when to have them.

RESPONDENT P02: I wish that I can be more involved as a father figure to the kids of my brother, they do not have a father figure, there is one who is 22 years old this year but she has 3 kids and all of them are first born and if I talk about a father figure I mean someone who will guide them whether they are in school or they are working.

INTERVIEWER: Which aspect or area do you think you need to work on, to become an involved father?

RESPONDENT P05: Time management, like I have mentioned that I need to work on my time and try to allocate more time for them instead of going out with a friend for drinks.

RESPONDENT P01: I think the areas that I need to tighten up on is that thing of being soft-hearted on the kids, generally, I love kids and I end up giving everyone the attention instead of giving my own more attention, my biological kids I do not stay with them, they are too far so I give all the love to the ones that I stay with and those around me in the township and the ones that should be getting all my attention they are not getting it.

RESPONDENT P09: I think that I need to strengthen the relationship with her mother because we do not see eye to eye with her, when she comes to collect her, she stands at the gate, and I fetch my kid from the house, she is troublesome, so what I need is to be able to co-parent with her. That what I need to work on.

INTERVIEWER: Okay, will move to the next set of questions, as fathers how do you take care of yourself?
RESPONDENT P12: I make sure that I take my treatment regularly, respect, and honouring God.

RESPONDENT P06: I take care of my diet and even the body, I exercise and eat fruits, I eat fruit salads and veggies.

RESPONDENT P10: one of the thing you should learn as a person is to be aligned with God, pray, so that when you go to the world you will be protected, tell him that God I know I do everything but please be with me and align me with your will, because you can eat and eat but if God is not watching you then your life is in danger

RESPONDENT P04: what I can say is that in everything that I do, I keep myself healthy, eating healthy, exercise, read books, having enough rest.

INTERVIEWER: what kind of support do you need to be able to take care of yourself?

RESPONDENT P5: I think the support that I need is a better partner, maybe someone I will be able to do things with and someone who can assist me.

RESPONDENT P08: the support I need to be honest with myself, I do not take care of my body but the support that I need I want to quit alcohol, to be honest alcohol is wasting my time and money, you

know I do not drink less than a thousand, the thousand looks small now, I do not start Thursday, I drink every day, so that is why I am saying I need support to stop.

INTERVIEWER: The question is, as fathers what encourages you to accompany your partner or children for healthcare services?

RESPONDENT P05: Well, I will say, what discourages me is that, when you go to the clinic with your partner, you find out that the nurse will come and ask you who is with this child, then the mother will say it is me, then they will ask you and "you my brother"? You answer and say I am with them then they ask you to go sit outside now she is alone inside the clinic, and I get bored outside, then next time she asks me to accompany her to the clinic I will refuse because I get bored. So, it discourages me. so, the staff attitude discourages us, so if you go to the facilities, you will find out that the women are inside, and all men are sitting outside.

RESPONDENT P05: the thing is you end up staying 4-5 hours outside so that discourages us a lot, obvious if it is just me and the baby its better because I am the older person who is with the baby, but

RESPONDENT P02: With me, I would go with them to the clinic for one reason, you know that women get excited and love seeing a man who likes babies, you go to the clinic, then you start getting attention from the sister nurse and end up getting her numbers.

INTERVIEWER: What can be done to support fathers who accompany their partners and/or children to health facilities?

RESPONDENT P08: Attention and respect

RESPONDENT P12: someone who will assist me as I come in, someone who is warmer in welcoming.

INTERVIEWER: What do you think will be acceptable and preferred incentives for accompanying their partners or children to the clinic?

RESPONDENT P05: sharing of experiences, If there might be a person that seats outside with you and keep your company, and maybe if they come back they can share their experiences, and interact with me, I think that might be better and maybe they can share something that can encourage you as the father, and they can share their own experiences so that I cannot go back angry and bored, I mean I will not have the correct information but it will not encourage me to become a better father.

RESPONDENT P09: for me, it would be better if I can get a guarantee of how much time she will spend in that facility because now, most men I know and work with have the mentality that women spend so much time in the clinic on purpose.

INTERVIEWER: how about material things that can be given to father?

RESPONDENT P12: Actually, we would like to see, those big screens we had before, which display some information for example if they are displaying circumcision, it keeps repeating one thing over and over again. So, if you are sitting there while you are listening to it even if you are not watching it but it sinks in your mind, it's like self-enforcement

RESPONDENT P09: I support P12, one thing about the fathers they do not like reading, there are pamphlets all over, but you will never see them reading those, fathers are lazy when they are in the clinic because most of them do not know how it works, they want to be assisted from one station to

another so with that thing as well, if men can understand how the clinics work unlike the mothers they understand how it works and they are strong than me going there.

RESPONDENT P03: real men do not read, that approach does not work, so if they can design those adverts and posters, in a way that will discourage us to do a bad thing but encourages us to do the right thing. Also, the language they use, sometimes you find out that those brochures are written in small handwriting that is a turnoff. At least it must have pictures then we can work with that.

RESPONDENT P12: I am saying time, if you say 2 hours, it must be 2 hours it shouldn't be more.

RESPONDENT P02: if I go to the clinic myself, I am talking from my experience, the nurse will ask me, what is wrong with me, and I will tell them that I have a headache, nurses must not stop there, they must probe and ask more questions, for instance, they must tell me about the prostate, because sometimes I see the posters and information about the prostate but I also want to hear her talking about it. if we can get a holistic assessment because I have seen the posters but let's deal with it clinically now. Another reason that makes us men not go to the clinic is the cultural factor if we can have male designated for us, for instance, if I come to the clinic I must also get a male nurse that will help me because you find a 70 years old man being attended by a woman and he needs to take off his clothes, and that nurse is the same age as your child, now you look at that nurse like your own child, so if we can have male nurses.

RESPONDENT P04: men being assisted by women is another issue of being naked, so we need more male clinicians someone who will understand what you are talking about because they have also maybe gone through the same thing.

RESPONDENT P09: I think I agree with the note that we need more male clinicians because that person can screen you everything entirely and holistically, other than when you get female clinicians, she will only concentrate on a specific point, she does not also look at anything outside than what you are there for, for instance, if you are coming to collect your medication, she will give you your medication and she is done with you. She does not go through other issues, e.g., prostate cancers and diabetics, if you find a man at least we can talk like the man to man, but at some point, we can relate man to man.
